# Differential Gene Expression in the Brain of the African Lungfish, *Protopterus annectens*, after Six Days or Six Months of Aestivation in Air

**DOI:** 10.1371/journal.pone.0071205

**Published:** 2013-08-16

**Authors:** Kum C. Hiong, Yuen K. Ip, Wai P. Wong, Shit F. Chew

**Affiliations:** 1 Natural Sciences and Science Education, National Institute of Education, Nanyang Technological University, Singapore, Republic of Singapore; 2 Department of Biological Sciences, National University of Singapore, Singapore, Republic of Singapore; Kyushu Institute of Technology, Japan

## Abstract

The African lungfish, *Protopterus annectens*, can undergo aestivation during drought. Aestivation has three phases: induction, maintenance and arousal. The objective of this study was to examine the differential gene expression in the brain of *P. annectens* during the induction (6 days) and maintenance (6 months) phases of aestivation as compared with the freshwater control using suppression subtractive hybridization. During the induction phase of aestivation, the mRNA expression of *prolactin* (*prl)* and *growth hormone* were up-regulated in the brain of *P. annectens*, which indicate for the first time the possible induction role of these two hormones in aestivation. Also, the up-regulation of mRNA expression of *tyrosine 3-monooxygenase/tryptophan 5-monooxygenase activation protein γ polypeptide* and the down-regulation of *phosphatidylethanolamine binding protein*, suggest that there could be a reduction in biological and neuronal activities in the brain. The mRNA expression of *cold inducible RNA-binding protein* and *glucose regulated protein 58* were also up-regulated in the brain, probably to enhance their cytoprotective effects. Furthermore, the down-regulation of *prothymosin α* expression suggests that there could be a suppression of transcription and cell proliferation in preparation for the maintenance phase. In general, the induction phase appeared to be characterized by reduction in glycolytic capacity and metabolic activity, suppression of protein synthesis and degradation, and an increase in defense against ammonia toxicity. In contrast, there was a down-regulation in the mRNA expression of *prl* in the brain of *P. annectens* during the maintenance phase of aestivation. In addition, there could be an increase in oxidative defense capacity, and up-regulation of transcription, translation, and glycolytic capacities in preparation for arousal. Overall, our results signify the importance of reconstruction of protein structures and regulation of energy expenditure during the induction phase, and the needs to suppress protein degradation and conserve metabolic fuel stores during the maintenance phase of aestivation.

## Introduction

Lungfishes are an archaic group of sarcopterygian fishes characterized by the possession of a lung opening off the ventral side of the esophagus. Sarcopterygians are recognized as “living fossils” whose evolutionary history dates back to the early Devonian period, some 390 million years ago. They hold an important position in the evolutionary tree with respect to water-land transition and their close phylogenetic relationships with tetrapods. There are six species of extant lungfishes, four of which (*Protopterus aethiopicus*, *Protopterus amphibius*, *Protopterus annectens* and *Protopterus dolloi*) can be found in Africa. African lungfishes are obligatory air-breathers; they typically inhabit fringing weedy areas of lakes and rivers where dissolved oxygen levels are low, daytime temperatures are high, and seasonal drying is common. During extended periods of drought, they enter into aestivation in mud cocoons [1], [2]. Aestivation involves corporal torpor at high environmental temperature with absolutely no intake of food and water for an extended period. Among the four African species, *P. annectens* is known to be the most dependent on aestivation; it normally aestivates for periods of 7–8 months in the wild, and a captive lungfish has emerged from its cocoon after periods of up to seven years [3], [4], [5], [6]. Recently, it has been reported that African lungfishes can be induced to aestivate in completely dried mucus cocoon in plastic boxes or in mud cocoon in the laboratory [7], [8], [9], [10], [11], [12].

There are three phases of aestivation: induction, maintenance and arousal [1]. As water dries up, the fish hyperventilates and secretes large amounts of mucus which turns into a dry mucus cocoon within 6–8 days. This period constitutes the induction phase of aestivation, during which, the African lungfish has to detect environmental cues and turn them into some sort of internal signals that would instill the necessary changes at the behavioral, structural, physiological and biochemical levels in preparation for the maintenance phase of aestivation. After entering the maintenance phase, the fish has to prevent cell death and degradation of biological structures. At the same time, it has to suppress the utilization of internal energy stores, and to sustain a slow rate of waste production in order to minimize pollution of the internal environment. Upon the return of favorable environmental conditions, the fish must arouse from aestivation, excrete the accumulated waste products, and feed for repair and growth. It can therefore be deduced that metabolic changes would vary in different phases of aestivation.

However, past research focused predominantly on the maintenance phase of aestivation, and there is a dearth of knowledge concerning molecular, biochemical and physiological mechanisms that are called into play during the induction phase. Only until very recently, did Loong et al. [11], [12] report on the differential gene expression, and the up-regulation of mRNA expression of carbamoyl phosphate synthetase III and ornithine-urea cycle capacity, in the liver of *P. annectens* during the induction phase (the first 6 days) of aestivation in air. More importantly, there are very few recent studies [13], [14] on the brain of aestivating African lungfish in spite of its possible role in coordinating a whole-body aestivation-specific response during the induction phase of aestivation. Therefore, this study was undertaken to examine, using suppression subtractive hybridization (SSH) polymerase chain reaction (PCR), the up- and down-regulation of gene expression in the brain of *P. annectens* during the induction phase (6 days) or the prolonged maintenance phase (6 months) of aestivation in air with reference to the freshwater control. It was hoped that results obtained would shed light on genes that were essential to the initiation, coordination and maintenance of the whole-body aestivation, and genes that were involved in the reduction in metabolism and the protection of biological structures in the brain of aestivating *P. annectens.*

## Materials and Methods

### Collection and Maintenance of Fish

*Protopterus annectens* (80–120 g body mass) were imported from Central Africa through a local fish farm in Singapore. They were maintained in plastic aquaria filled with dechlorinated freshwater at pH 7.0 and at 25°C in the laboratory. Water was changed daily. No attempt was made to separate the sexes. Fish were acclimated to laboratory conditions for at least 1 month before experimentation. During the adaptation period, fish were fed with frozen fish meat and food was withheld 96 h prior to experiments. Approval to undertake this study was obtained from the Institutional Animal Care and Use Committee of the National University of Singapore (IACUC 035/09).

### Experimental Conditions and Tissue Sampling

*Protopterus annectens* were induced to aestivate at 27–29°C and 85–90% humidity individually in plastic tanks (L29 cm×W19 cm×H17.5 cm) containing 15 ml of dechlorinated tap water (adjusted to 0.3‰ with seawater) following the procedure of Chew et al. [7]. During the induction phase of aestivation, the experimental fish would secrete plenty of mucus during the first few days, and the mucus would slowly dry up between day 5 and day 7 to form a mucus cocoon. Aestivation was considered to begin when the fish was fully encased in the cocoon and displayed no locomotor activities. *Protopterus annectens* can be maintained in aestivation for a long period of time and this was regarded as the maintenance phase of aestivation.

Fish maintained in freshwater served as controls. Control fish were killed with an overdose of neutralized MS222 (0.2%) followed with a blow to the head. Aestivating fish were killed on day 6 (end of induction phase or beginning of maintenance phase) or day 186 (6 months; prolonged maintenance phase) with a blow to the head. The brain was quickly excised and frozen in liquid nitrogen. The frozen samples were kept at −80°C until analysis.

### Total RNA and Poly (A) mRNA Extraction

Frozen tissues were homogenized using a polytron homogenizer (Kinematica AG, Lucerne, Switzerland) in 400 µl of chaotropic buffer (4.5 M guanidine thiocyanate, 2% N-lauroylsarcosine, 50 mM EDTA (pH 8.0), 25 mM Tris-HCl (pH 7.5), 0.1 M β-mercaptoethanol, 0.2% antifoam A). Total RNA was extracted from the brain, using the chaotropic extraction protocol described by Whitehead and Crawford [15]. The RNA pellet obtained was rinsed twice with 500 µl of 70% ethanol, and further purified using the Qiagen RNeasy Mini Kit (Qiagen Inc., Valencia, CA, USA). The concentration and purity of the purified RNA were determined using the NanoDrop ND-1000 spectrophotometer (Thermo Fisher Scientific Inc., Wilmington, DE, USA). The RNA quality was determined by visualising the presence of the 18S and 28S ribosomal RNA bands using the Bio-Rad Universal Hood II gel documentation system (Bio-Rad, Hercules, CA, USA) after carrying out electrophoresis of 1 µg of RNA on 1% (w/v) agarose gel in TAE buffer (40 mM Tris-acetate, 1 mM EDTA, pH 8.0) with nucleic acid staining dye GelRed (1∶20000, Biotium Inc., Hayward, CA, USA) at 100 V for 30 min. The presence of sharp 28S and 18S bands in the proportion of about 2∶1 indicate the integrity of the total RNA.

Poly (A) mRNA was extracted from 200 µg of total RNA using the Oligotek mRNA kit (Qiagen Inc.). The RNA sample (200 µg) was mixed with 15 µl of Oligotex suspension (resin) and was heated at 70°C for 3 min and then cooled at 25°C for 10 min. The Oligotex:mRNA complex was spun at 14,000×*g* and the pellet obtained was resuspended in 400 µl of Buffer OW2 (Qiagen Inc.) and then passed through a small spin column by centrifuging at 14,000×*g* for 1 min. The column was washed with another 400 µl of Buffer OW2. The resin in the column was resuspended with 50 µl of hot (70°C) Buffer OEB (Qiagen Inc.) and eluted by centrifugation at 14,000×*g* for 1 min to obtain the Poly (A) RNA. Another 50 µl of hot (70°C) Buffer OEB was added to the column and the process was repeated to ensure maximal Poly (A) mRNA yield.

### Construction of SSH Libraries

Two sets of forward (up-regulated genes) and reverse (down-regulated genes) SSH libraries for the brain were generated using the PCR-Select™ cDNA subtraction kit (Clontech Laboratories, Inc., Mountain View, CA, USA); one set for fish aestivated for 6 days in air (induction phase) with reference to the freshwater control, and the other set for fish aestivated for 6 months in air (prolonged maintenance phase) with reference to the freshwater control. Two micrograms of poly (A) mRNA from each condition was used for cDNA synthesis. After the first and second strand synthesis, the double stranded cDNA from both groups were digested with Rsa I. A portion of the digested cDNA was ligated with either Adapter 1 or Adaptor 2R, and the rest was saved for subsequent usage as the driver for hybridization. The forward library was generated from the hybridization between adapter-ligated cDNA obtained from fish that had undergone 6 days or 6 months of aestivation in air (tester) and Rsa I-digested cDNA from the control fish kept in freshwater (driver). The reverse library was made the same way, except that the adapter-ligated cDNA from the control in freshwater served as the tester while the Rsa I-digested cDNA from fish aestivated for 6 days or 6 months of aestivation in air acted as the driver. The driver cDNA was added in excess to remove common cDNA by hybrid selection, leaving over-expressed and novel tester cDNAs to be recovered and cloned. The PCR amplification of the differentially expressed cDNAs was performed using the Advantage cDNA polymerase mix (Clontech Laboratories, Inc.) and 9902 Applied Biosystems PCR thermal cycler (Life Technologies Corporation, Carlsbad, CA, USA). The primary and secondary PCR amplification of these reciprocal subtractions of cDNA from the control and aestivated fish produced 1 forward and 1 reverse SSH libraries enriched in differentially expressed transcripts.

Differentially expressed cDNAs were cloned using pGEM®-T easy vector system kit (Promega Corporation, Madison, WI, USA), transformed into chemically competent JM109 *Escherichia coli* (Promega Corporation), and plated onto Luria-Bertani (LB) agar with ampicillin, 5-bromo-4-chloro-3-indolyl-β-D-galactopyranoside (X-gal) and isopropyl β-D-thiogalactopyranoside (IPTG). Selected white colonies were grown overnight in LB broth with ampicillin. The plasmids were extracted using the resin-based plasmid miniprep kit (Axygen Biosciences, Union City, CA, USA). The plasmids were quantified by the NanoDrop ND-1000 spectrophotometer. Approximately 80–100 ng of plasmid DNA was used in BigDye® Terminator v3.1 Cycle Sequencing Kit (Life Technologies Corporation) with 2 µM T7 primers. Excess fluorescent nucleotides and salts were removed from the samples by ethanol precipitation. The dried samples were resuspended in Hi-Di Formamide (Life Technologies Corporation) before loading to the Prism™ 3130XL sequencer (Life Technologies Corporation). A total of 500 clones for each forward and reverse library were selected for sequencing.

Sequence output was exported as text and edited manually to remove vector sequences using BioEdit Sequence Alignment Editor software version 7.0.9 [16]. BLAST searches were performed using the tBLASTx algorithm [17] and default search conditions. Proteins were considered significant when the *E* value was <1E-04. The annotated sequences were grouped based on Gene Ontology classification. The zebrafish nomenclature system (see https://wiki.zfin.org/display/general/ZFINZebrafishNomenclatureGuidelines) for genes and proteins of fish origin and the human nomenclature (see http://www.genenames.org/guidelines.html) for genes and proteins of mammalian origin were adopted in this paper.

### Relative Quantitative Real-time PCR (qPCR)

In order to validate the changes obtained in the SSH studies, seven genes were selected for the determination of mRNA expression using quantitative real-time PCR (qPCR). These include *pyruvate kinase* (*pk*), *fumarate hydratase* (*fh*), *glutamine synthetase* (*gs*), *phosphofructokinase* (*pfk*), *prolactin* (*prl*), *Na^+^/K^+^-ATPase α2* (*nkaα2*) and *ferritin heavy chain* (*fth*). Prior to first strand cDNA synthesis, RNA from the brain of fish kept in freshwater, aestivated for 6 days in air or aestivated for 6 months in air were treated separately with Deoxyribonuclease I (Qiagen Inc.) to remove any contaminating genomic DNA. First strand cDNA was synthesized from 1 µg of total RNA using random hexamer primer and the RevertAid™ first stand cDNA synthesis kit, following the manufacturer’s instruction (Thermo Fisher Scientific Inc). The mRNA expression of selected genes were quantified using a StepOnePlus™ Real-Time PCR System (Life Technologies Corporation). Each PCR reaction contained 5 µl of 2x Fast SYBR ® Green Master Mix (Life Technologies Corporation), a certain aliquot of gene-specific primers (listed in Table 1) and 0.1–2 ng of cDNA in a total volume of 10 µl. Samples were run in triplicate. qPCR reactions were performed with the following cycling conditions: 95°C for 20 s (1 cycle), followed by 40 cycles of 95°C for 3 s and 60°C of 30 s. Data was collected at each elongation step. Each run was followed by a melt curve analysis by increasing the temperature from 60°C to 95°C at 0.3°C increment to confirm the presence of only a single PCR product. In addition, random PCR products were electrophoresed in a 1.8% agarose gel to verify that only one band was present. All the data were normalized to the abundance of *β-actin* mRNA. The amplification efficiencies for *β-actin* and all selected genes were between 90–100%. The subsequent application of the 2^−ΔΔCT^ calculation for relative quantification was validated by confirming that the variation between the amplification efficiencies of the target and reference gene through a 100-fold dilution remained relatively constant [18]. The mean fold-change values were transformed into logarithmic values (log_2_) to enable valid statistical analysis.

**Table 1:** Primers used for quantitative real-time PCR on *fumarate hydratase* (*fh*), *ferritin heavy chain* (*fth*), *glutamine synthetase* (*gs*), *Na^+^/K^+^-ATPase α2* (*nkaα2*), *phosphofructokinase* (*pfk*), *pyruvate kinase* (*pk*) and *prolactin* (*prl*), with *β-actin* as the reference gene, from the brain of *Protopterus annectens*.

Gene	Primer sequence (5′ to 3′)
*fh* (JZ347458)	Forward (5′-TAGTAACAGCACTCAACCCAC-3′)
	Reverse (5′-GCTTGACCCACTGATCAAACTG-3′)
*fth* (JZ347374)	Forward (5′-CTCAGGTCCGCCAGAACTA-3′)
	Reverse (5′-GCCACATCATCTCGGTCAA-3′)
*gs* (JZ347462)	Forward (5′-GTGACATGTACCTCATCCCA-3′)
	Reverse (5′-TACTCCTGCTCCATGCCAAACCA-3′)
*nkaα2* (JZ347474)	Forward (5′-AGACATTGCAGCACGTCTC-3′)
	Reverse (5′-CATTCATTTCCTTCAAATCCGAA-3′)
*pfk* (JZ347479)	Forward (5′-TTTGCCAACACCGTAGATACA-3′)
	Reverse (5′-GCACAAAGTCAGTCTCGTCT-3′)
*pk* (JZ347493)	Forward (5′-GCGTGGTGACTTGGGTATAG-3′)
	Reverse (5′-CGAGTTGGACGTGGCTTT-3′)
*prl* (JZ347487)	Forward (5′-CAACTGTCATACCTCATCACTG-3′)
	Reverse (5′-CTTCATTACTAGCCGCAAGAG-3′)
*β-actin*	Forward (5′-CATACTGTGCCCATTTATGAAGGT-3′)
	Reverse (5′-CAAGTCACGGCCAGCTAAATC-3′)

### Statistical Analysis

Results for qPCR were presented as means ± standard errors of the mean (S.E.M.). Student’s t-test was used to evaluate the difference between means. Differences with *P*<0.05 were regarded as statistically significant.

## Results

### Induction Phase (6 days) of Aestivation

Two subtracted libraries, forward (Table 2) and reverse (Table 3), were constructed to determine the genes that were up- and down-regulated, respectively, in the brain of *P. annectens* which had undergone 6 days of aestivation (induction phase) in air. A total of 130 genes were identified from these subtraction libraries. Interestingly, many more genes were up-regulated (80 genes; Table 2) than down-regulated (50 genes; Table 3) in the brain of *P. annectens* after 6 days of aestivation. There were 570 unidentified sequences which could be genes that have yet to be characterized in *P. annectens*. *Tubulin alpha 4a* (*tuba4a*) and some *ribosomal protein* mRNAs appeared in both forward and reverse subtraction libraries, indicating that they could be false positives or they encode for different isoforms of the same protein.

**Table 2:** Known transcripts found in the forward library (up-regulation) obtained by suppression subtractive hybridization PCR from the brain of *Protopterus annectens* aestivated for 6 days in air with fish kept in freshwater as the reference for comparison.

Group and Gene	*P. annectens* accession no.	Biological processes	E-value	No of clones
**Apoptosis**				
Plasminogen activator inhibitor 1 RNA-binding protein	JZ347480	Regulation of anti-apoptosis	3.00E-07	1
**Carbohydrate metabolism**				
Enolase	JZ347451	Glycolysis	4.00E-31	1
Fructose-bisphosphate aldolase C	JZ347457	Glycolysis	4.00E-91	3
Fumarate hydratase	JZ347458	TCA cycle	5.00E-30	2
Pyruvate kinase	JZ347493	Glycolysis	9.00E-35	1
**Cell cycle and proliferation**				
BRCA2 and CDKN1A interacting protein	JZ347437	Cell cycle and DNA repair	2.00E-57	1
Growth hormone precursor	JZ347464	Positive regulation of growth	0	2
RAN, member RAS oncogene family	JZ347497	Cell cycle	3.00E-173	1
Secreted acidic cysteine rich glycoprotein	JZ347521	Regulation of cell proliferation	1.00E-65	4
**Lipoprotein, fatty acid and cholesterol homeostasis** **and transport**				
c11orf2 homolog	JZ347439	Lipid transport	8.00E-56	1
**Ion binding and transport**				
ADP/ATP translocase 2 putative	JZ347428	Membrane transport	8.00E-50	5
Mitochondrial ATP synthase beta subunit	JZ347472	ATP synthesis	9.00E-35	5
Sec61 beta subunit	JZ347520	Membrane transport	1.00E-64	2
Solute carrier family 20, member 1b	JZ347524	Phosphate transport	3.00E-17	1
Solute carrier family 25 alpha, member 5	JZ347526	Membrane transport	1.00E-51	1
**Iron metabolism and transport**				
Apoferritin higher subunit	JZ347429	Iron binding	2.00E-88	1
**Nitrogen metabolism**				
Glutamine synthetase	JZ347462	Glutamine biosynthetic process	2.00E-23	2
**Nucleic acid binding and transcription**				
Brain abundant, membrane attached signal protein 1	JZ347436	Negative regulation of gene-specific transcription	8.00E-18	2
Breast carcinoma amplified sequence 2	JZ347438	RNA splicing	1.00E-83	2
Ctr9, Paf1/RNA polymerase II complex component	JZ347445	Histone monoubiquitination	0	1
DEAH (Asp-Glu-Ala-His) box polypeptide 15	JZ347449	RNA splicing	6.00E-07	1
H3 histone, family 3B	JZ347379	Nucleosome assembly	3.00E-74	1
High mobility group protein-1	JZ347466	Positive regulation of transcription	9.00E-16	1
Histone H2A.Z putative mRNA	JZ347467	Nucleosome assembly	3.00E-97	3
p68 RNA helicase	JZ347477	ATP dependent helicase activity	7.00E-06	4
Poly(A) binding protein, cytoplasmic 1	JZ347481	RNA splicing	2.00E-54	2
Polymerase (RNA) II (DNA directed) polypeptide J	JZ347482	Transcription	2.00E-36	3
Small nuclear ribonucleoprotein E	JZ347523	RNA splicing	2.00E-64	2
SRA stem-loop-interacting RNA-binding protein, mitochondrial precursor	JZ347527	Regulation of transcription	8.00E-38	1
Tripartite motif protein 28	JZ347537	Regulation of transcription	5.00E-19	1
Y box binding protein 1 isoform 2	JZ347547	mRNA splicing	6.00E-61	2
**Protein degradation**				
Proteasome (prosome macropain) subunit alpha type 4	JZ347489	Proteolysis	5.00E-95	1
**Protein synthesis, transport and folding**				
40S ribosomal protein S2	JZ347424	Translation	3.00E-65	2
40S ribosomal protein S30	JZ347425	Translation elongation	8.00E-37	4
Amyloid beta (A4) precursor protein	JZ347354	Protein transport	6.00E-48	5
Eukaryotic translation elongation factor 1 alpha 2	JZ347371	Translation	4.00E-25	40
Eukaryotic translation elongation factor 1 beta 2	JZ347452	Translation	9.00E-54	1
Eukaryotic translation initiation factor 3, subunit 3 gamma	JZ347454	Translation	2.00E-28	1
Lipocalin	JZ347384	Protein transport	1.00E-23	28
Prefoldin subunit 2	JZ347483	Protein binding	1.00E-31	1
RAB36, member RAS oncogene family	JZ347495	Protein transport	5.00E-11	1
Ribosomal protein L12	JZ347505	Translation	7.00E-74	5
Ribosomal protein L3 fragment 1	JZ347500	Translation	2.00E-35	2
Ribosomal protein L35	JZ347509	Translation	7.00E-41	2
Ribosomal protein L41	JZ347510	Translation	3.00E-21	1
Ribosomal protein L4-like	JZ347502	Translation	5.00E-133	2
Ribosomal protein L5	JZ347503	Ribosome assembly	0	1
Ribosomal protein L7a-like	JZ347392	Ribosome biogenesis	9.00E-78	8
Ribosomal protein S12	JZ347516	Translation	3.00E-36	2
Ribosomal protein S27a	JZ347519	Translation	5.00E-97	3
Ribosomal protein S2e	JZ347511	Translation	1.00E-75	1
Ribosomal protein S3	JZ347512	Translation	0	2
Ribosomal protein S4	JZ347514	Translation	0	8
Transmembrane protein 11	JZ347536	Protein binding	2.00E-17	2
**Response to stimulus**				
Cold-inducible RNA-binding protein	JZ347442	Response to stress	4.00E-33	3
Ganglioside-induced differentiation-associated protein 1-like 1	JZ347460	Response to retinoic acid	2.00E-29	2
**Signaling**				
Prolactin	JZ347487	Lactation, positive regulation of cell proliferation	5.00E-14	15
RAC/CDC42 exchange factor	JZ347496	Regulation of Rho protein signal transduction	2.00E-39	1
RAS-like, family 11, member B	JZ347498	Signal transduction	1.00E-25	2
Reticulon 1-C.1	JZ347499	Signal transduction	2.00E-04	1
**Structural**				
Tubulin alpha-1 chain putative	JZ347538	Cell structure	9.00E-170	9
Tubulin, alpha 4a	JZ347416	Cell structure	0	11
Tubulin, beta	JZ347540	Cell structure	7.00E-44	1
Tubulin, beta 5	JZ347541	Cell structure	5.00E-164	1
**Others**				
ATG7 autophagy related 7 homolog	JZ347431	Autophagy	2.00E-44	1
Cardiac muscle alpha actin 1	JZ347440	Unclassified	2.00E-67	1
Cell cycle associated protein 1b	JZ347441	Unclassified	2.00E-12	2
Creatine kinase, mitochondrial 1	JZ347444	ATP binding	3.00E-150	1
Dynamin 1-like	JZ347450	GTP catabolic process	2.00E-26	6
Fumarylacetoacetate hydrolase	JZ347459	Cellular amino acid metabolic process	8.00E-10	2
Glucose regulated protein, 58 kDa	JZ347461	Unclassified	1.00E-40	1
HMP19 protein	JZ347468	Unclassified	6.00E-43	2
Myosin, light chain 1, alkali; skeletal, fast,	JZ347473	Muscle contraction	4.00E-27	1
Nasopharyngeal epithelium specific protein 1	JZ347475	Unclassified	1.00E-46	2
Ornithine decarboxylase antizyme	JZ347476	Cell differentiation	2.00E-137	3
Prolyl 4-hydroxylase, alpha polypeptide II	JZ347488	Unclassified	1.00E-25	2
Quinoid dihydropteridine reductase	JZ347494	Metabolic process	2.00E-19	1
Stromal cell derived factor receptor 1	JZ347529	Unclassified	1.00E-39	6
Tyrosine 3-monooxygenase/tryptophan 5-monooxygenase activation protein, gamma polypeptide	JZ347542	Protein targeting	1.00E-54	2
Ubiquinol-cytochrome c reductase core protein I	JZ347543	Electron transport chain	8.00E-55	2

**Table 3:** Known transcripts found in the reverse library (down-regulation) obtained by suppression subtractive hybridization PCR from the brain of *Protopterus annectens* aestivated for 6 days in air with fish kept in freshwater as the reference for comparison.

Group and Gene	*P. annectens* accession no.	Biological processes	E-value	No of clones
**Carbohydrate metabolism**				
Glyceraldehyde-3-phosphate dehydrogenase	JZ347463	Glycolysis	6.00E-22	1
Phosphofructokinase	JZ347479	Glycolysis	4.00E-163	7
**Cell cycle and proliferation**				
Protein phosphatase 1 (*pp1*) gamma 1	JZ347490	Cell cycle	2.00E-172	11
SUMO-conjugating enzyme UBC9	JZ347531	Cell cycle	4.00E-147	5
**Cell structure**				
Actin, gamma 1	JZ347426	Cell structure	3.00E-70	4
Transmembrane protein 2	JZ347535	Integral to membrane	1.00E-49	2
Tubulin, alpha 4a	JZ347539	Microtubule–based process	9.00E-16	1
UPF0466 protein C22orf32, mitochondrial	JZ347545	Integral to membrane	1.00E-12	3
**Complement**				
Complement component factor H	JZ347443	Complement activation	6.00E-07	2
**DNA repair and protection**				
Activity-dependent neuroprotector homeobox	JZ347427	Neuroprotection	2.00E-16	3
Apoptosis-inducing, TAF9-like domain 1	JZ347430	DNA repair	2.00E-16	1
**Ion binding and transport**				
ATP synthase, H^+^ transporting, mitochondrial F_0_ complex, subunit c-3	JZ347432	ATP synthesis coupled proton transport	1.00E-41	1
ATP synthase, H^+^ transporting, mitochondrial F_0_ complex, subunit f	JZ347433	ATP synthesis coupled proton transport	6.00E-33	2
ATPase, Na^+^/K^+^ transporting, beta 1a polypeptide	JZ347434	Ion transport	1.00E-09	1
Na^+^/K^+^-ATPase alpha 2 subunit	JZ347474	Cation transport	0	3
Solute carrier family 25 (mitochondrial carrier; phosphate carrier), member 3	JZ347525	Transmembrane transport	1.00E-30	2
Voltage-dependent anion-selective channel protein 2	JZ347546	Anion transport	8.00E-132	1
**Iron, copper metabolism and transport**				
Beta-2-globin	JZ347435	Oxygen transport	7.00E-05	2
**Lipoprotein, fatty acid and cholesterol homeostasis** **and transport**				
Lipoyltransferase 1	JZ347471	Protein lipoylation	9.00E-27	3
Sulfotransferase family 4A, member 1	JZ347530	Lipid metabolic process	1.00E-72	1
**Nucleic acid binding and transcription**				
KH domain containing, RNA binding, signal transduction associated 1b	JZ347469	RNA binding	3.00E-05	5
Protein phosphatase 1A magnesium-dependent, alpha isoform	JZ347491	Regulation of transcription	4.00E-79	1
Prothymosin, alpha	JZ347492	Transcription	4.00E-24	13
Staufen 1	JZ347528	Intracellular mRNA localization	2.00E-35	3
Transcription elongation factor B polypeptide 2	JZ347533	Regulation of transcription	6.00E-40	1
Translin	JZ347534	DNA binding	3.00E-71	1
**Protein degradation**				
Ubiquitin conjugating enzyme E2	JZ347544	Protein degradation	2.00E-90	9
Serpin peptidase inhibitor, clade B (ovalbumin),member 1, like 3	JZ347522	Serine type endopeptidase inhibitor activity	1.00E-08	1
**Protein synthesis, transport and folding**				
Ribosomal protein L3 fragment 2	JZ347501	Translation	2.00E-28	1
DEAD (Asp-Glu-Ala-Asp) box polypeptide 21	JZ347448	Ribosomal RNA biogenesis, editing, transport	4.00E-05	4
Eukaryotic translation elongation factor 2	JZ347453	Translation	2.00E-39	1
Eukaryotic translation initiation factor 4E-binding protein 2	JZ347455	Translation initiation	4.00E-09	9
Procollagen C-endopeptidase enhancer 2	JZ347486	Heparin/protein binding	1.00E-23	1
Ribosomal protein L22-like 1	JZ347506	Translation	4.00E-79	1
Ribosomal protein L31	JZ347507	Translation	3.00E-88	3
Ribosomal protein L34	JZ347508	Translation	6.00E-88	1
Ribosomal protein L7a-like	JZ347504	Ribosome biogenesis	2.00E-101	3
Ribosomal protein S13	JZ347517	Ribosome assembly	5.00E-105	1
Ribosomal protein S20	JZ347518	Translation	6.00E-85	1
Ribosomal protein S3A	JZ347513	Translation	5.00E-146	1
Ribosomal protein S9	JZ347515	Translation	4.00E-122	10
**Response to stimulus**				
Cyclic AMP-regulated phosphoprotein, 21 kD	JZ347446	Cellular response to heat	4.00E-22	9
FK506 binding protein 1B, 12.6 kDa	JZ347456	Regulation of heart rate, response to glucose stimulus	7.00E-68	4
**Signaling**				
Guanine nucleotide binding protein (G protein), alpha activating activity polypeptide O	JZ347465	G-protein coupled receptor protein signaling pathway	2.00E-04	1
Phosphatidylethanolamine binding protein	JZ347478	Regulation of mitosis and signaling	4.00E-52	1
**Others**				
Cytochrome c oxidase subunit VIa polypeptide 1	JZ347447	Oxidation reduction	1.00E-27	14
KH domain-containing transcription factor B3	JZ347470	Unclassified	4.00E-10	3
Prepromesotocin	JZ347484	Unclassified	5.00E-32	1
Preprosomatostatin 2	JZ347485	Regulation of cell migration	3.00E-164	5
Telomere associated repeat sequence	JZ347532	Unclassified	2.00E-20	1

Results obtained from the forward library (Table 2) demonstrated for the first time that the mRNA expression of certain genes related to signaling (*prl*), cell cycle and proliferation [*growth hormone* (*gh*)], and transcription in general were up-regulated in the brain of *P. annectens* after 6 days of aestivation in air. A number of ribosomal genes that was involved in protein synthesis were also up-regulated. Other up-regulated genes included those involved in carbohydrate metabolism [*enolase* (*eno*), *fructose-bisphosphate aldolase C* (*aldoc*), *fh* and *pk*], nitrogen metabolism (*gs*) and iron metabolism.

The fewer genes that were down-regulated in the brain of *P. annectens* after 6 days of aestivation in air (Table 3) included genes involved in cell cycle, protein synthesis, protein degradation, ion binding and transport [*nkaα2* and *Na^+^/K^+^-ATPase β1a* (*nkaβ1a*)], and oxidation reduction [*cytochrome c oxidase subunit VIa polypeptide 1* (*cox6a1*)]. There was also a down-regulation in the mRNA expression of some genes related to carbohydrate metabolism [*pfk* and *glyceraldehyde 3-phosphate dehydrogenase* (*gapdh*)].

### Maintenance Phase (6 Months) of Aestivation

Forward (Table 4) and reverse (Table 5) libraries were also constructed to reflect the genes that were up- and down-regulated, respectively, in the brain of *P. annectens* after 6 months of aestivation in air. Unlike the brain of *P. annectens* that had undergone 6 days of aestivation in air, only a total of 81 genes were identified from these subtraction libraries. Again, the forward library showed that more (63) genes were up-regulated (Table 4) while the reverse library showed that only 18 genes were down-regulated (Table 5). Out of the 1000 sequences obtained, 458 sequences were unidentified and they could again be genes that have yet to be characterized in *P. annectens*. *Ribosomal protein L19* appeared in both forward and reverse subtraction libraries, indicating that it could be a false positive or it could be encoding for different isoforms of the same protein in the 2 libraries.

**Table 4:** Known transcripts found in the forward library (up-regulation) obtained by suppression subtractive hybridization PCR from the brain of *Protopterus annectens* aestivated for 6 months in air with fish kept in freshwater as the reference for comparison.

Group and Gene	*P. annectens*accession no.	Biological processes	E-value	No of clones
**Antioxidant**				
Superoxide dismutase	JZ347414	Antioxidant	5.00E-34	1
**Apoptosis**				
Tumor protein, translationally-controlled 1	JZ347418	Anti-apoptosis	2.00E-53	2
**Carbohydrate metabolism**				
Alpha enolase-1	JZ347353	Glycolysis	1.00E-48	1
Enolase 2	JZ347369	Glycolysis	2.00E-71	4
Glyceraldehyde-3-phosphate dehydrogenase	JZ347377	Glycolysis	5.00E-161	4
Pyruvate kinase	JZ347390	Glycolysis	1.00E-56	4
**Cell structure**				
Actin-related protein 2/3 complex subunit 3	JZ347351	Actin polymerization	4.00E-131	2
Beta actin	JZ347358	Cell structure	1.00E-174	1
Calponin 3, acidic b	JZ347359	actomyosin structure organization	1.00E-09	1
Cofilin-2 putative	JZ347362	Cell structure	3.00E-18	1
Myosin light polypeptide 6 putative	JZ347385	Muscle filament sliding	7.00E-19	1
Tubulin beta-1 chain putative	JZ347415	Microtubule-based movement	2.00E-50	1
Tubulin, alpha 4a	JZ347416	Microtubule-based movement	0	1
Type I keratin isoform 1	JZ347419	Cell structure	1.00E-06	8
Up-regulated during skeletal muscle growth protein 5	JZ347420	Integral to membrane	8.00E-26	2
**Ion binding and transport**				
Adenine nucleotide translocase	JZ347352	ADP/ATP transport	6.00E-129	4
Calretinin putative mRNA	JZ347360	Calcium ion binding	3.00E-68	1
Cysteine and histidine-rich domain (CHORD)-containing,zinc binding protein 1	JZ347363	Calcium ion bindng	1.00E-28	2
voltage-dependent anion channel 2	JZ347421	Anion transport	2.00E-69	2
voltage-dependent anion channel 3	JZ347422	Anion transport, synaptictransmission	5.00E-72	4
**Iron metabolism and transport**				
Ferritin, middle subunit	JZ347375	Cellular iron ion homeostasis,iron ion transport	3.00E-57	1
**Lipoprotein, fatty acid and cholesterol homeostasis** **and transport**				
Apolipoprotein O	JZ347355	Lipid transport	1.00E-22	1
**Nucleic acid binding and transcription**				
H3 histone, family 3B	JZ347379	Nucleosome assembly	1.00E-79	4
H3 histone, family 3C	JZ347380	Nucleosome assembly	8.00E-64	1
High mobility group B3b	JZ347381	DNA binding	1.00E-05	1
Histone H3.3 putative	JZ347382	Nucleosome assembly	1.00E-61	1
Polymerase (RNA) II (DNA directed) polypeptide G	JZ347389	Transcription	4.00E-143	1
Splicing factor, arginine/serine-rich 11	JZ347413	RNA splicing	6.00E-13	1
**Protein degradation**				
Dipeptidylpeptidase 8	JZ347364	Proteolysis	2.00E-39	2
**Protein synthesis, transport and folding**				
Amyloid beta (A4) precursor protein	JZ347354	Protein transport	3.00E-69	2
Elongation factor 1-alpha putative	JZ347366	Translational elongation	2.00E-78	1
Elongation factor 1-beta putative	JZ347367	translational elongation	3.00E-93	2
Eukaryotic translation elongation factor 1 alpha	JZ347372	Translational elongation	2.00E-75	3
Eukaryotic translation elongation factor 1 alpha 1	JZ347370	Translational elongation	4.00E-91	7
Eukaryotic translation elongation factor 1 alpha 2	JZ347371	Translational elongation	3.00E-107	32
Eukaryotic translation elongation factor 1 gamma	JZ347373	Translational elongation	2.00E-27	3
Glycyl-tRNA synthetase	JZ347378	Glycyl-tRNA aminoacylation, translation	9.00E-68	1
Lipocalin	JZ347384	Protein transport	1.00E-23	7
Ribosomal protein L10	JZ347394	Translation	2.00E-40	1
Ribosomal protein L11	JZ347395	Translation	4.00E-130	6
Ribosomal protein L12	JZ347505	Translation	6.00E-102	13
Ribosomal protein L13	JZ347396	Translation	2.00E-76	3
Ribosomal protein L19 fragment 1	JZ347397	Translation	4.00E-104	2
Ribosomal protein L21	JZ347399	Translation	3.00E-79	2
Ribosomal protein L23	JZ347400	Translation	1.00E-130	5
Ribosomal protein L26	JZ347401	Ribosome large subunit biogenesis	6.00E-32	1
Ribosomal protein L41	JZ347510	Translation	3.00E-04	10
Ribosomal protein L7a-like	JZ347392	Ribosome biogenesis	1.00E-104	2
Ribosomal protein Large P0	JZ347404	Translational elongation	2.00E-165	1
Ribosomal protein P2	JZ347405	Translational elongation	4.00E-73	2
Ribosomal protein S12	JZ347410	Translation	2.00E-40	5
Ribosomal protein S20	JZ347411	Translation	1.00E-35	5
Ribosomal protein S23	JZ347412	Translation	7.00E-49	5
Ribosomal protein S3	JZ347406	Translation	0	2
Ribosomal protein S4	JZ347514	Translation	0	3
Ribosomal protein S5 putative	JZ347407	Translation	2.00E-21	1
Ribosomal protein S7	JZ347408	Translation	0	4
Ribosomal protein S8	JZ347409	Translation	2.00E-84	1
Ribosomal protein S9	JZ347515	Translation	0	1
**Response to stimulus**				
Cold-inducible RNA binding protein	JZ347442	Response to stress	3.00E-29	9
FK506 binding protein 1B, 12.6 kDa	JZ347456	Regulation of heart rate, response to glucose stimulus	1.00E-12	4
**Others**				
Ribosomal protein 5S-like protein	JZ347391	Unclassified	1.00E-54	12
XMAB21	JZ347423	Unclassified	2.00E-33	1

**Table 5:** Known transcripts found in the reverse library (down-regulation) obtained by suppression subtractive hybridization PCR from the brain of *Protopterus annectens* aestivated for 6 months in air with fish kept in freshwater as the reference for comparison.

Group and Gene	*P. annectens* accession no.	Biological processes	E-value	No of clones
**Cell structure**				
Beta-tubulin	JZ347417	Cell structure	2.00E-67	1
Myristoylated alanine-rich protein kinase C substrate	JZ347386	Cell structure	2.00E-10	3
**Ion binding and transport**				
ATPase, H^+^ transporting, V0 subunit C	JZ347356	ATP synthesis coupledproton transport	4.00E-25	4
Basic leucine zipper and W2 domains 1	JZ347357	Regulation of transcription	2.00E-05	1
**Iron metabolism and transport**				
Ferritin heavy chain	JZ347374	Cellular iron ion homeostasis,iron ion transport	2.00E-75	1
**Protein synthesis, transport and folding**				
Coatomer protein complex, subunit gamma 2	JZ347361	Intracellular protein transport,vesicle-mediated transport	7.00E-23	104
DnaJ (Hsp40) homolog, subfamily A, member 1	JZ347365	Protein folding	4.00E-33	1
Elongation factor-1, delta, b	JZ347368	Translation elongation	4.00E-63	1
Ribosomal protein L19 fragment 2	JZ347398	Translation	2.00E-82	3
Ribosomal protein L36a	JZ347402	Translation	2.00E-62	2
Ribosomal protein L38	JZ347403	Translation	1.00E-32	1
Ribosomal protein L8	JZ347393	Translation	6.00E-112	3
**Signaling**				
Prolactin	JZ347487	lactation, positive regulation ofcell proliferation	1.00E-20	43
**Others**				
Gephyrin	JZ347376	Establishment of synaptic specificityat neuromuscular junction	0	1
HN1-like protein	JZ347383	Unclassified	2.00E-05	4
KH domain-containing transcription factor B3	JZ347470	Unclassified	4.00E-08	146
NADH dehydrogenase 1 alpha subcomplex subunit 13	JZ347387	Electron transport chain	4.00E-45	3
Peptidyl-glycine alpha-amidating monooxygenase A precursor	JZ347388	Peptide metabolic process,oxidation reduction	3.00E-28	1

Unlike the induction phase, some genes involved in protein synthesis, iron metabolism, carbohydrate metabolism (e.g., *pk*), and lipid metabolism appeared in the forward library (Table 4) indicating that their mRNA expression were up-regulated in the brain of *P. annectens* after 6 months of aestivation in air. As for the reverse library (Table 5), genes that were down-regulated included *prl* and others involved in cell structure, iron metabolism (e.g., *fth*), and nucleic acid binding.

### Confirmation of Up- or Down-regulation of Selected Genes using qPCR

**Figure 1:**
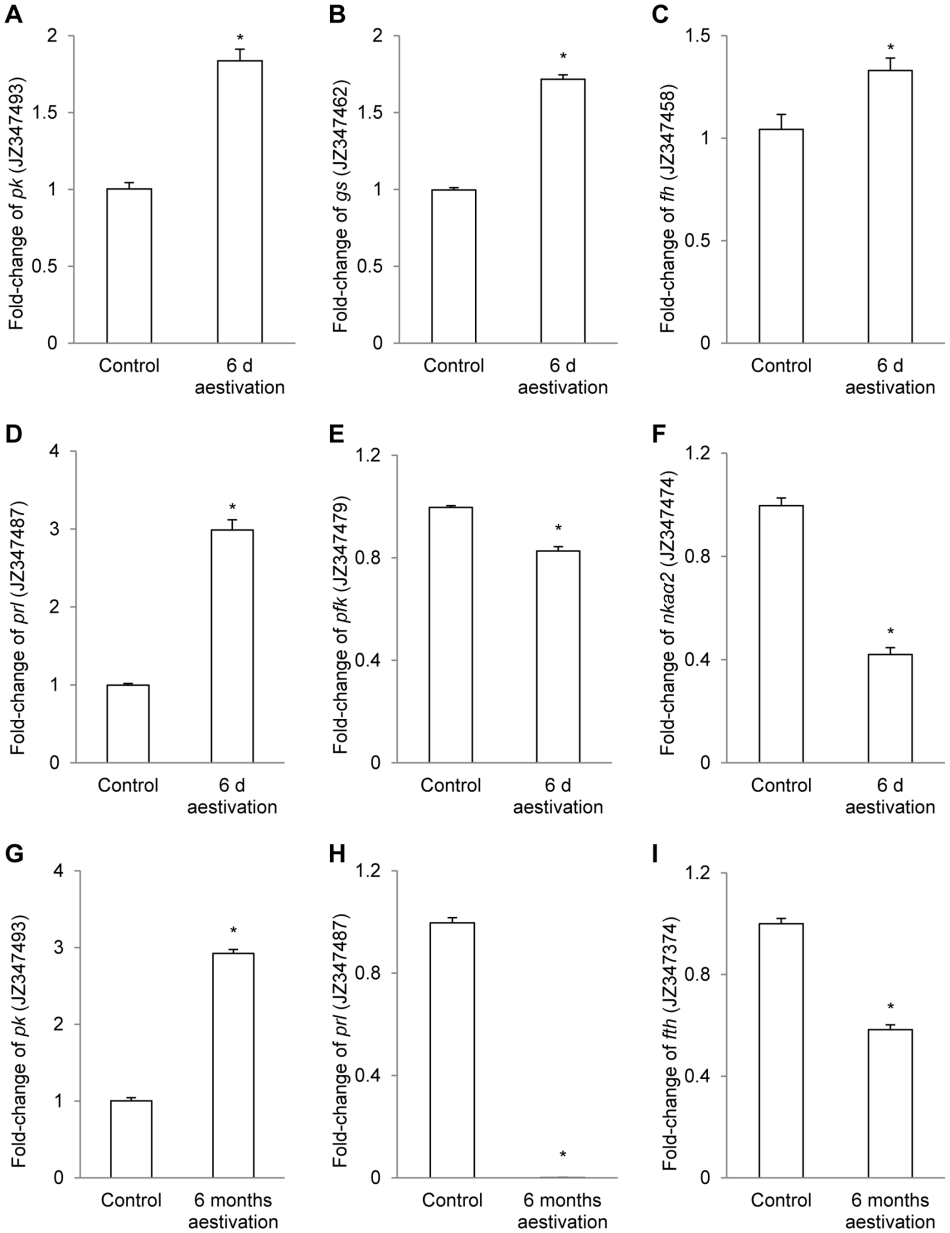
qRT-PCR of selected genes that were differentially expressed based on suppression subtractive hybridization. Relative quantification of mRNA expression (fold change) of (A) *pyruvate kinase* (*pk*, JZ347493), (B) *glutamine synthetase* (*gs*, JZ347462), (C) *fumarate hydratase* (*fh*, JZ347458), (D) *prolactin* (*prl*, JZ347487), (E) *phosphofructokinase* (*pfk*, JZ347479), (F) *Na^+^/K^+^-ATPase α2* (*nkaα2*, JZ347474), (G) *pyruvate kinase* (*pk*, JZ347493), (H) *prolactin* (*prl*, JZ347487) and (I) *ferritin heavy chain* (*fth*, JZ347374), using β-actin as the reference gene, in the brain of *Protopterus annectens* aestivated for 6 days (d) (A–F) or 6 months (G–I) in air with reference to the those of fish kept in freshwater as control. Results represent mean+S.E.M. (*N* = 6). *Significantly different from the corresponding freshwater control (*P*<0.05).

In agreement with the SSH results of the brain of *P. annectens* after 6 days of aestivation in air, there were significant increases in the mRNA expression of *pk*, *gs*, *fh*, and *prl* (Fig. 1A–D) and significant decreases in the mRNA expression of *pfk* (Fig. 1E) and *nkaα2* (Fig. 1F). Also, there was a significant increase in the mRNA expression of *pk* (Fig. 1G) in the brain of *P. annectens* after 6 days of aestivation in air and significant decreases in the mRNA expression of *prl* (Fig. 1H) and *fth* (Fig. 1I) in corroboration of the SSH results.

## Discussion

### More Genes are Up-regulated than Down-regulated during the Induction and Maintenance Phases of Aestivation

Surprisingly, many more genes were up-regulated than down-regulated in the brain of *P. annectens* during the induction and maintenance phases of aestivation, although one would expect exactly the opposite in a situation of metabolic down-regulation [19]. From the physiological point of view, aestivation has often been traditionally associated with metabolic depression [20], because conservation of metabolic fuels has been regarded as an important adaptation during long periods of aestivation without food intake. Furthermore, strong global suppression of gene expression is an integral part of metabolic rate depression in various hypometabolic systems that have been studied to date [19]. However, while the association between aestivation and metabolic depression is clearly present in endothermic mammals during aestivation, it is debatable whether it can be universally applied to aestivating ectothermic animals [1]. For instance, whether metabolic depression in turtles is an adaptation to aestivation per se or simply a response to fasting [21], [22] remains an open question. In fact, the decrease in oxygen consumption in laboratory-aestivating yellow mud turtle, *Kinosternon flavescens*, is identical to that of fully hydrated turtles that are fasted for an equivalent period [23], [24]. Based on studies mainly on *P. aethiopicus*, it has long been accepted that a profound decrease in metabolic rate occurs in African lungfishes in general during the maintenance phase of aestivation in a mud cocoon or an artificial substratum [25], [26], without reference to whether aestivation takes place in hypoxia or normoxia. However, Perry et al. [27] reported that *P. dolloi* exhibited constant rates of O_2_ consumption before (0.95±0.07 mmol kg^−1^ h^−1^), during (1.21±0.32 mmol kg^−1^ h^−1^) and after (1.14±0.14 mmol kg^−1^ h^−1^) extended periods (1–2 months) of aestivation in a completely dried mucus cocoon in air (normoxia). Subsequently, Loong et al. [10] obtained results which suggested that metabolic depression in aestivating African lungfish was triggered by hypoxia and not an integral part of aestivation.

In the past, the occurrence of organic structural modifications in aestivating animals has been largely neglected, but to date, aestivation in African lungfishes are known to be associated with structural and functional modifications in at least the heart and the kidney [28], [29]. Icardo et al. [28] reported that the myocytes in the trabeculae associated with the free ventricular wall of *P. dolloi* showed structural signs of low transcriptional and metabolic activity (heterochromatin, mitochondria of the dense type) while in water [28]. These signs are partially reversed in aestivating fish (euchromatin, mitochondria with a light matrix), and paradoxically, aestivation appears to trigger an increase in transcriptional and synthetic myocardial activities, especially at the level of the ventricular septum [28]. In addition, Ojeda et al. [28] demonstrated structural modifications in all the components of the renal corpuscle of aestivating *P. dolloi*. These changes can be reversed after arousal, indicating that the renal corpuscle is a highly dynamic structure capable of modifying its architecture in response to different phases of aestivation. Morphological down-regulation and quick restoration of morphology during the maintenance phase and arousal phase, respectively, also occur in the intestine of *P. annectens* [30]. Thus, aestivation cannot be regarded as the result of a general depression of metabolism, but it involves the complex interplay between up-regulation and down-regulation of diverse cellular activities, and aestivation would logically involve variations in rates of protein degradation and protein synthesis, reconstructing and regenerating cells and tissues during the induction and arousal phases, respectively, through a rapid protein turnover. Such a proposition is corroborated by the fact that a greater variety of genes were up-regulated than down-regulated in the brain of *P. annectens* during the induction and maintenance phases of aestivation. Whether increased mRNA expression would lead to increases in protein expression through increased translational activities is unclear at present.

### Induction Phase: Prl and Gh could be Involved in Inducing and Coordinating Aestivation

PRL, GH and somatolactin are three pituitary hormones whose genes are considered to have evolved from a common ancestral gene [31]. PRL affects a number of physiological processes and among them are the control of mammary gland development, initiation and maintenance of lactation, immune modulation, osmoregulation, control of hypothalamic releasing-inhibiting factors, and behavioral modification. At the cellular level, PRL exerts mitogenic, morphogenic, and secretory activities. In fish, the major function of Prl is osmoregulation in freshwater to prevent the loss of Na^+^ [32], and in terrestrial adaptation [33]. The up-regulation in the mRNA expression of *prl* in the brain of *P. annectens* during the induction phase of aestivation (Table 2) indicate for the first time that Prl might have an important role in the aestivation process, although the mechanism is unknown at present.

A number of endogenous hormonal substances have been proposed to be involved in initiating and maintaining aestivation [34]. These substances are often related to sleep e.g., Gh, somatostatin and arginine vasotocin. In human, GH secretion was associated with sleep and the maximal GH levels occur within minutes of the onset of slow wave sleep [35]. Somatostatin is a cyclic tetradecapeptide that acts as a negative regulator for the secretion of growth hormone. It is synthesized as a large precursor molecule before it is processed into its active form. In the brain of the European hamster, *Cricetus cricetus*, somatostatin levels were reported to be significantly lower in hibernating winter specimens than euthermic winter animals [36]. The novel findings on the down-regulation of *preprosomatostatin 2* (Table 3), together with the up-regulation of *gh* (Table 2) in the brain of *P. annectens* during the induction phase of aestivation indicate the importance of these genes in the induction of aestivation.

### Induction Phase: Reduction in Biological Activity of the Brain

The tyrosine 3-monooxygenase/tryptophan 5-monooxygenase activation protein (14-3-3), β polypeptide, γ polypeptide and ζ polypeptide are highly abundant in the brain. They are activators of tyrosine and tryptophan hydroxylases, which are also known as tyrosine 3-monooxygenase and tryptophan 5-monooxygenase, respectively [37]. These two hydroxylases are the initial and rate-limiting enzymes in the biosynthesis of dopamine and serotonin, respectively. Both dopamine and serotonin were involved in regulating sleep- waking cycle [38], [39]. Moreover, through interaction with more than 100 binding partners, these proteins are crucial for various physiological cellular processes such as signaling, cell growth, division, adhesion, differentiation and apoptosis [37]. The up-regulation of the mRNA expression of *tyrosine 3-monooxygenase/tryptophan 5-monooxygenase activation protein γ polypeptide* (*ywhaγ*) in the brain of *P. annectens* during 6 days of aestivation (Table 2) might lead to the increased production of dopamine and serotonin, which might be important in regulating the biological activity of the brain.

Phosphatidylethanolamine binding protein (PEBP) is a signaling protein that plays a role in neurotransmission. In mammals, PEBP modulates the signaling pathways by regulating the activation of mitogen-activated protein kinase (MAPK), nuclear factor of kappa light polypeptide gene enhancer in B cells (NF-κB) and also the G-protein coupled receptors [40]. The most important brain-specific function of PEBP elucidated to date is its role as precursor for hippocampal cholinergic neurostimulating peptide, which in mammals is known to stimulate the activity and the growth of acetylcholine neurons in the brain [40], [41]. The down-regulation of *pebp* in the brain of *P. annectens* after 6 days of aestivation (Table 3) suggested that there could be a reduction in neuronal activity.

### Induction Phase: Cytoprotection through Increased Expression of Cold Inducible RNA-binding Protein and Glucose Regulated Protein 58

The cold inducible RNA-binding protein (CIRBP) is a member of a glycine-rich RNA binding protein family. It is known to be induced by cold stress [42]. In vitro studies have shown that the CIRBP has cytoprotective effects [43] and regulates neural development. Using cultured neural stem cells, Saito and co-workers [44] reported an increase in mRNA expression for *CIRBP* and the protein abundance at 32°C compared to those cultured at 37°C. In addition, the knockdown of this gene by RNA interference in neural stem cells showed an increase in apoptotic cell population, indicating the cytoprotective effect of this protein [44]. The up-regulation of *cirbp* mRNA was also reported when salmon was subjected to hyperosmotic stress during the transition from freshwater to marine environment [45]. Since *P. annectens* would encounter osmotic and dehydration stresses during the induction and maintenance phase of aestivation, the up-regulation of the mRNA expression of *cirbp* could enhance its cytoprotective effects in the brain of *P. annectens* during the aestivation process.

Glucose regulated protein 58 (GRP58) is a member of the protein disulfide isomerase family of proteins that are present mainly, but not exclusively, in the endoplasmic reticulum (ER). It was first identified as a stress protein in response to glucose deprivation and its well-characterized functions include acting as a molecular chaperone to help in the correct folding of glycoprotein [46] and the assembly of the major histocompatibility complex class 1. GRP58 serves as a carrier protein in Alzheimer’s disease to prevent the aggregation of β-amyloids [47]. Hence, the up-regulation of *grp58* in the brain of *P. annectens* during the induction phase (6 days) of aestivation (Table 2) may prevent misfolding of proteins to preserve biological structures.

### Induction Phase: Suppression of Proliferation of Brain Cells by Down-regulation of Prothymosin α

Prothymosin α (PTMAα) is a ubiquitously and abundantly expressed small nuclear protein [48], [49], [50] that is involved in cell proliferation [51] and protection against apoptosis [52], [53]. Over-expression of PTMAα accelerates cell proliferation [54], [55], whereas inhibition of PTMAα synthesis prevents cell division [56] and induces apoptosis [57]. Consistent with its properties, PTMAα is particularly abundant in tumor cells [58], [59]. Mounting evidence suggests PTMAα involvement in transcription regulation [60], [61], [62]. Thus, the down-regulation of the mRNA expression of *ptmaα* (Table 3) in the brain of *P. annectens* during the induction phase of aestivation suggests that there could be a suppression of transcription and cell proliferation which is an essential preparation for the maintenance phase of aestivation.

### Induction Phase: A Reduction in Glycolytic Capacity

During the induction phase of aestivation, the mRNA expression of several genes related to carbohydrate metabolism, e.g., *eno*, *aldoc* and *pk* were up-regulated (Table 2), but there were also down-regulation in the mRNA expression of *gapdh* and *pfk* (Table 3) which happened to be the regulatory enzyme of glycolysis. The precise regulation of PFK prevents glycolysis and gluconeogenesis from occurring simultaneously. Therefore judging by changes in the mRNA expression of *pfk*, which in turns affects one of the regulatory steps in the glycolytic pathway, it would appear that there could be a decrease in the glycolytic capacity in the brain of *P. annectens* during the induction phase of aestivation. The changes in the mRNA expression of *pk* might indicate the needs to prevent an increase in gluconeogenesis in relation to a decrease in glycolysis, since the balance between these two processes would be controlled by Pk and phosphoenolpyruvate carboxykinase over the conversion between phosphoenolpyruvate and pyruvate.

The down-regulation in the mRNA expression of *gapdh* in the brain of *P. annectens* during the induction phase is consistent with the proposition that there could be a decrease in the glycolytic capacity. However, Gapdh has multiple functions in mammals and some of these may extend to fish. Zheng et al. [63] reported that GAPDH could itself activate transcription. The *octamer binding protein coactivator in S phase* (*OCA-S*) transcriptional coactivator complex contains GAPDH. GAPDH moves between the cytosol and the nucleus and may thus link the metabolic state to gene transcription [63]. Hara et al. [64] showed that GAPDH initiates apoptosis. GAPDH also appears to be involved in the vesicle transport from the ER to the Golgi apparatus which is part of the shipping route for secreted proteins [65]. Hence, a down-regulation in the mRNA expression of *gapdh* might also indicate a suppression of transcription, apoptosis and protein secretion, which could be regarded as adaptive responses, in the brain of *P. annectens* during the induction phase of aestivation.

### Induction Phase: Reduction in Metabolic Activity

At present, there is a dearth of knowledge concerning metabolism of the brain of African lungfishes during aestivation, especially concerning the substrates used to supply energy for various metabolic processes. Even if there is a high glycogen store in the brain, it is a limited source of energy store. Hence, it is logical to deduce that the brain would still have to depend on glucose supply from the blood during aestivation. However, aestivating lungfish would have a slower heart beat rate [26], [66], and hence a slow rate of blood circulation. Consequently, it would be essential for the brain to reduce its metabolic rate, including the rate of ATP synthesis through oxidative phosphorylation. Indeed, the down-regulation of ATP synthases indicates that ATP synthesis was reduced during aestivation. In general, metabolic rate reduction requires a coordinated suppression of the rate of cellular ATP turnover, including both ATP-generating and ATP-consuming reactions. Hence, the down-regulation of mRNA expressions of *nkaα2*- and *nkaβ1a-* subunits and *voltage-dependent anion channel* (*vdac*) 2 (*vdac2*) in the brain of *P. annectens* during the induction phase of aestivation (Table 3) corroborates the proposition that a reduction in metabolic activities in the brain could be essential to aestivation.

### Induction Phase: Suppression of Protein Synthesis and Degradation

Protein synthesis is a major energy expense in cells and it will utilise up to 50% of the cellular energy during translation [67], [68], [69]. Although down-regulation of protein synthesis is a consistent phenomenon in organisms undergoing metabolic depression, translation of genes must become more selective in order to conserve energy but at the same time, allows critical functions to carry on during the subsequent maintenance phase of aestivation. A number of genes related to protein synthesis, transport and folding appeared in the forward and reverse libraries of *P. annectens* after 6 days of aestivation. Besides various ribosomal proteins, the mRNA expression of *eukaryotic translation elongation factor* (*eef) 1α2* (*eef1α2*; 40 clones) and *eef1β2* (1 clone), and *eef3γ3* (1 clone) were up-regulated (Table 2), while those of *eef2* (1 clone) and *eukaryotic translation initiation factor 4E-binding protein 2* (9 clones) were down-regulated (Table 3), instead. Overall, these results indicate there could be simultaneous up- and down-regulation in the synthesis of certain proteins. This could be important for the brain to make the necessary preparation to enter into the maintenance phase of aestivation without an over expenditure of energy [1].

SUMO enzymatic cascade catalyzes the dynamic posttranslational modification process of SUMOylation. SUMOylation is a post-translational modification involved in various cellular processes, such as nuclear-cytosolic transport, transcriptional regulation, apoptosis, protein stability, response to stress, and progression through the cell cycle [70].http://en.wikipedia.org/wiki/SUMO_protein - cite_note-0 A reduction of SUMO-conjugating enzyme (UBC9) prevents cell cycle progression at the G2 or early M phase, causing the accumulation of large budded cells with a single nucleus, a short spindle and replicated DNA in *Xenopus* oocyte [71]. Protein phosphatase 1 (PP1) belongs to a certain class of phosphatases known as protein serine/threonine phosphatases, which control glycogen metabolism, muscle contraction, cell progression, neuronal activities, splicing of RNA, mitosis [72], cell division, apoptosis, protein synthesis, and regulation of membrane receptors and channels [73]. Therefore, the down-regulation of SUMO-conjugating enzyme *ubc9* and *pp1* suggests that there could be a suppression of protein degradation in the brain of *P. annectens* during the induction phase of aestivation. Only then, would there be preservation of protein structure and function during the aestivation process. In addition, the down-regulation of these two genes may be important in arresting the cell cycle and growth during the early stage of aestivation. Together, these adaptive responses would reduce energy expenditure and conserve the limited endogenous energy reserve.

### Induction Phase: Defense Against Ammonia Toxicity

Gs catalyzes the formation of glutamine from ammonia and glutamate and the reaction requires ATP, and it is essential for the detoxification of ammonia to glutamine in fish [74], [75]. Neural tissues are sensitive to ammonia, and thus Gs activity is high in most fish brains [76], [77], [78], [79]. Four *gs* isoforms have been identified in the rainbow trout *Oncorhynchus mykiss* [80], which are differentially expressed in the brain and liver of fish exposed to elevated environmental ammonia [81]. At present, it is uncertain whether Gs isoforms are present in African lungfishes. However, it is logical to deduce that Gs could be up-regulated in the brain of *P. annectens* to detoxify ammonia produced within the brain during aestivation. This is especially important considering the fact that aestivating lungfish would have a decrease in heart beat rate [26], [66], and hence a decrease in blood circulation might slow down the removal of ammonia that was produced endogenously within the brain. Although, it is highly unlikely that amino acids were degraded for energy production in the brain; rather, it is probable that ammonia would be released from amino acid catabolism due to an increased turnover of protein synthesis and protein degradation during the induction phase to prepare the brain to enter into the maintenance phase of aestivation.

### Maintenance Phase: Prolactin could be involved in Maintaining Whole-body Aestivation

In contrast to the induction phase, there was a down-regulation in the mRNA expression of *prl* in the brain of *P. annectens* during the prolonged maintenance phase of aestivation (Table 5). A total of 43 clones (out of 500) of *prl* appeared in the reverse library, indicating that there could be a drastic suppression of *prl* expression which was supported by qPCR results. This corroborates the proposition that Prl could be an important part of the signaling mechanisms to induce and maintain aestivation in *P. annectens*.

### Maintenance Phase: Superoxide Dismutase was Involved in Oxidative Defense in the Brain

Increased synthesis and/or activity of intracellular antioxidant enzymes can protect cellular macromolecules from potentially lethal stress-induced damage. The brain of hibernating squirrel is characterized by increased oxidative stress resistance [82], [83], [84]. Over-expression of superoxide dismutase (SOD), both mitochondrial (MnSOD) and cytosolic (CuZnSOD), increases oxidative stress resistance [85], [86], [87]. In addition, over-expression of SODs, glutathione peroxidase (GPx) and catalase (CAT) also act to protect against ischemia associated cytochrome c release from mitochondria, thereby limiting the occurrence of apoptotic cell death [88]. Similarly, Page et al. [89] found that most of the intracellular antioxidant enzymes, including the MnSOD, CuZnSOD, CAT, GPx and glutathione reductase were up-regulated in brain tissue of lungfish that had aestivated for 60 days. Therefore, Page et al. [89] concluded that aestivating *P. dolloi* had enhanced oxidative stress resistance in the brain due to a significant up-regulation of intracellular antioxidant capacity. Results obtained from this study are in agreement with the conclusion of Page et al. [89] because there was an up-regulation of the mRNA expression of *sod1* in the brain of *P. annectens* during the prolonged maintenance phase (6 months) of aestivation (Table 4).

### Maintenance Phase: Regulation of ATP Usage through an Up-regulation of *vdac2*

VDACs are pore-forming proteins found in the outer mitochondria membrane of eukaryote. They are the major channels for metabolites to permeate the outer mitochondrial membrane. In mammals, they are known to play an essential role in cellular metabolism. They conduct ATP to bind to several cytosolic carbohydrate kinases, e.g. hexokinase, glycerol kinase and creatine kinase, and thus providing bound kinases with preferential access to mitochondrial ATP [90]. Hence, the up-regulation of *vdac2* and *vdac3* in the brain of *P. annectens* after 6 months of aestivation (Table 4) may reflect on the limited ATP supply by the mitochondria and the importance of transporting ATP to essential kinases on the outer mitochondria membrane to facilitate its usage by the relatively more important metabolic pathways.

### Maintenance Phase: Increased Glycolytic and Protein Synthesis Capacities in Preparation for Arousal?

Six months of aestivation led to increases in mRNA expression of several enzymes involved in glycolysis (*eno1*, *eno2*, *gapdh* and *pk*) in the brain of *P. annectens* (Table 4). Unlike the induction phase, both *gapdh* and *pfk* no longer appeared in the reverse library. Taken together, these results indicated that the glycolytic capacity in the brain of *P. annectens* varied between the induction and maintenance phases. Simply based on the SSH results, it is difficult to draw a definitive conclusion, but it would appear that there was an up-regulation of the glycolytic capacity during the prolonged maintenance phase of aestivation. Considering that the aestivating fish was in a state of torpor and the importance of conserving endogenous energy stores, this could be viewed as a strategy in preparation for arousal, and an increase in glycolytic capacity might not imply an increase in glycolytic rate.

In comparison with the induction phase, more genes related to protein synthesis, transport and folding appeared in the forward library for lungfish that had entered into the prolonged maintenance phase of aestivation. Specifically, 2 *elongation factor 1* (*ef1*) genes (*ef1α* and *ef1β*), and 4 *eef1* genes (*eef1α*, *eef1α1*, *eef1α2* and *eef1γ*) were up-regulated, with 32 clones of *eef1α2* appearing in the forward library (Table 4). The increases in mRNA expression of these genes indicate that there could be an increase in the capacity for protein synthesis in the brain of *P. annectens*. This does not necessarily imply an increase in protein synthesis in situ, since the mRNA expression of *coatomer protein complex subunit gamma 2* (*copγ2*) was down-regulated with 104 clones (Table 5). Biosynthetic protein transport from the ER via the Golgi apparatus to the plasma membrane is mediated by vesicular carriers [91], [92], [93]. These vesicles, termed COPI-coated vesicles contain a fuzzy protein coat whose major coat protein components are ADP-ribosylation factor and coatomer, which is a protein complex made up of seven subunits [94], [95]. Thus, a down-regulation of the mRNA expression of *copγ2* suggested that the transport of protein, even if synthesized, was put on hold during the prolonged maintenance phase of aestivation, probably in preparation for arousal.

### Maintenance Phase: Up-regulation of Histone Expression

Histones are the chief protein components of chromatin, acting as spools around which DNA winds, and play a role in gene regulation. Five major families of histones (H) exist, and H3 is one of the core histones [96], [97]. Acetylation and methylation of different lysine (Lys) and arginine residues in H3 has been linked to either transcriptionally active or transcriptionally repressed states of gene expression [98], whereas phosphorylation of H3 was initially linked to chromosome condensation during mitosis [99], [100]. The phosphorylation of H3 at serine 10 (Ser 10) has an important role in the transcriptional activation of eukaryotic genes in various organisms. Methylation of Lys9 in H3 (H3K9) is a prominent modification that has been implicated in diverse processes, including transcriptional silencing, heterochromatin formation, and DNA methylation [101]. H3 also plays a crucial role in activating the spindle assembly checkpoint in response to a defect in mitosis [102]. Therefore, the up-regulation of mRNA expression of *h3* in the brain of *P. annectens* after 6 months of aestivation (Table 4) indicates that there could be an increase in the capacity of transcription, which could be an adaptive response for increased mitosis for tissue repair upon arousal.

### Conclusion

We have demonstrated by SSH PCR that during the induction phase of aestivation, several genes in the brain of *P. annectens* such as *prl*, *gh* and *preprosomatostatin 2* could be involved in inducing and coordinating aestivation. There could be a reduction in biological activities of the brain as an up-regulation of *ywhaγ* and a down-regulation of *pebp* in the brain were observed during the induction phase of aestivation. Our results also revealed that several gene clusters were up- or down-regulated in the brain of *P. annectens* after 6 days of aestivation in air. These aestivation-specific genes could be involved in a reduction in glycolytic capacity, metabolic activities and suppression of protein synthesis and degradation. By contrast there was a down-regulation of *prl* expression in the brain during the maintenance phase, corroborating the proposition that Prl plays an important role in coordinating aestivation in *P. annectens*. There was also an up-regulation of *sod1* expression in the brain during the maintenance phase of aestivation. Sod is important for oxidative defense, and oxidative defence could be important for life maintenance during the maintenance phase of aestivation. Furthermore, there could be an increase in glycolytic and protein synthesis capacities during the maintenance phase of aestivation, and the up-regulation of *h3* indicated an increase in capacity of transcription, which could be an adaptive response in preparation for the arousal phase of aestivation.

## References

[1] Ip YK, Chew SF (2010) Nitrogen metabolism and excretion during aestivation. In: Navas CA, Carvalho JE, editors. Progress in molecular and subcellular biology Vol 49, *Aestivation: Molecular and Physiological Aspects*. Berlin: Springer-Verlag. 63–94. 10.1007/978-3-642-02421-4_4 20069405

[2] Ballantyne JS, Frick NT (2010) Lungfish Metabolism. In: Jorgensen JM, Joss J, editors. The biology of lungfishes. New Hampshire: Science Publishers. 305–340.

[3] Smith HW (1931) Observations on the African lungfish, *Protopterus aethiopicus*, and on evolution from water to land environments. Ecology 12: 164–181.

[4] Greenwood PH (1986) The natural history of African lungfishes. J Morphol Suppl 1: 163–179.

[5] Lomholt JP (1993) Breathing in the aestivating African lungfish, *Protopterus amphibicus*. In: Singh BR, editor. Advances in Fish Research: Narendra, New Delhi. 17–34.

[6] Johnels AG, Svensson GSO (1954) On the biology of *Protopterus annectens* (Owen). Arkiv Zoologi 7: 131–164.

[7] Chew SF, Chan NK, Loong AM, Hiong KC, Tam WL, et al (2004) Nitrogen metabolism in the African lungfish (*Protopterus dolloi*) aestivating in a mucus cocoon on land. J Exp Biol 207: 777–786. 14747410 10.1242/jeb.00813

[8] Ip YK, Yeo PJ, Loong AM, Hiong KC, Wong WP, et al (2005) The interplay of increased urea synthesis and reduced ammonia production in the African lungfish *Protopterus aethiopicus* during 46 days of aestivation in a mucus cocoon. J Exp Zool A 303: 1054–1065. 10.1002/jez.a.237 16254918

[9] Loong AM, Hiong KC, Lee SML, Wong WP, Chew SF, et al (2005) Ornithine-urea cycle and urea synthesis in African lungfishes, *Protopterus aethiopicus* and *Protopterus annectens*, exposed to terrestrial conditions for six days. J Exp Zool 303A: 354–365. 10.1002/jez.a.147 15828011

[10] Loong AM, Pang CYM, Hiong KC, Wong WP, Chew SF, et al (2008) Increased urea synthesis and/or suppressed ammonia production in the African lungfish, *Protopterus annectens*, during aestivation in air or mud. J Comp Physiol B 178: 351–363. 18058110 10.1007/s00360-007-0228-6

[11] Loong AM, Hiong KC, Wong WP, Chew SF, Ip YK (2012) Differential gene expression in the liver of the African lungfish, *Protopterus annectens*, after 6 days of aestivation in air. J Comp Physiol B 182: 231–245. 21915614 10.1007/s00360-011-0613-z

[12] Loong AM, Chng YR, Chew SF, Wong WP, Ip YK (2012) Molecular characterization and mRNA expression of carbamoyl phosphate synthetase III in the liver of the African lungfish, *Protopterus annectens*, during aestivation or exposure to ammonia. J Comp Physiol B 182: 367–379. 22038021 10.1007/s00360-011-0626-7

[13] Giusi G, Crudo M, Di Vito A, Facciolo RM, Garofalo F, et al (2011) Lungfish aestivating activities are locked in distinct encephalic gamma-aminobutyric acid type A receptor alpha subunits. J Neurosci Res 89: 418–428. 21259328 10.1002/jnr.22553

[14] Giusi G, Zizza M, Facciolo RM, Chew SF, Ip YK, et al (2012) Aestivation and hypoxia-related events share common silent neuron trafficking processes. BMC Neurosci 13: 39. 22520032 10.1186/1471-2202-13-39 PMC3407487

[15] Whitehead A, Crawford DL (2005) Variation in tissue-specific gene expression among natural populations. Genome Biol 6: R13. 15693942 10.1186/gb-2005-6-2-r13 PMC551533

[16] Hall TA (1999) BioEdit: a user-friendly biological sequence editor and analysis program for Windows 95/98/NT. Nucleic Acids Symp Ser 41: 95–98.

[17] Altschul SF, Gish W, Miller W, Myers EW, Lipman DJ (1990) Basic local alignment search tool. J Mol Biol 215: 403–410. 2231712 10.1016/S0022-2836(05)80360-2

[18] Livak KJ, Schmittgen TD (2001) Analysis of relative gene expression data using real-time quantitative PCR and the 2(-Delta Delta C(T)) Method. Methods 25: 402–408. 11846609 10.1006/meth.2001.1262

[19] Storey KB, Storey JM (2010) Metabolic regulation and gene expression during aestivation. In: Navas CA, Carvalho JE, editors. Progress in molecular and subcellular biology Vol 49, *Aestivation: Molecular and Physiological Aspects*. Berlin: Springer-Verlag. 25–45. 10.1007/978-3-642-02421-4_2 20069403

[20] Storey KB (2002) Life in the slow lane: molecular mechanisms of estivation. Comp Biochem Physiol A 133: 733–754. 10.1016/s1095-6433(02)00206-4 12443930

[21] Belkin DA (1968) Aquatic respiration and underwater survival of two freshwater turtle species. Respir Physiol 4: 1–14. 5639522 10.1016/0034-5687(68)90002-9

[22] Sievert LM, Sievert GA, Cupp PV (1988) Metabolic rate of feeding and fasting juvenile midland painted turtles, *Chrysemys picta marginata* . Comp Biochem Physiol 90A: 157–159. 10.1016/0300-9629(88)91022-5 2900100

[23] Seidel ME (1978) Terrestrial dormancy in the turtle *Kinosternon flavescens*: respiratory metabolism and dehydration. Comp Biochem Physiol 61A: 1–4.

[24] Hailey A, Loveridge JP (1997) Metabolic depression during dormancy in the African tortoise *Kinixys spekii* . Can J Zool 75: 1328–1335.

[25] Smith HW (1930) Metabolism of the lungfish *Protopterus aethiopicus* . J Biol Chem 88: 97–130.

[26] Delaney RG, Lahiri S, Fishman AP (1974) Aestivation of the African lungfish *Protopterus aethiopicus*: cardiovascular and respiratory functions. J Exp Biol 61: 111–128. 4411892 10.1242/jeb.61.1.111

[27] Perry SF, Euverman R, Wang T, Loong AM, Chew SF, et al (2008) Control of breathing in African lungfish (*Protopterus dolloi*): a comparison of aquatic and cocooned (terrestrialized) animals. Resp Physiol Neurobiol 160: 8–17. 10.1016/j.resp.2007.06.015 17974507

[28] Icardo JM, Amelio D, Garofalo F, Colvee E, Cerra MC, et al (2008) The structural characteristics of the heart ventricle of the African lungfish *Protopterus dolloi*: freshwater and aestivation. J Anat 213: 106–119. 18482286 10.1111/j.1469-7580.2008.00901.x PMC2526117

[29] Ojeda JL, Wong WP, Ip YK, Icardo JM (2008) The renal corpuscle of the African lungfish *Protopterus dolloi*: Structural, histochemical and immunofluorescence modification during aestivation. Anat Rec 291: 1156–1172. 10.1002/ar.20729 18521897

[30] Icardo JM, Loong AM, Colvee E, Wong WP, Ip YK (2012) The alimentary canal of the African lungfish *Protopterus annectens* during aestivation and after arousal. Anat Rec (Hoboken) 295: 60–72. 21964967 10.1002/ar.21476

[31] Rand-Weaver M, Kawauchi H (1993) Growth hormone, prolactin and somatolactin: a structural overview. In: Hochachka PW, Mommsen TP, editors. Biochemistry and molecular biology of fishes: Elsevier Science Publishers, Amsterdam. 39–56.

[32] Ayson FG, Kaneko T, Tagawa M, Hasegawa S, Grau EG, et al (1993) Effects of acclimation to hypertonic environment on plasma and pituitary levels of two prolactins and growth hormone in two species of tilapia, *Oreochromis mossambicus* and *Oreochromis niloticus* . Gen Comp Endocrinol 89: 138–148. 8428646 10.1006/gcen.1993.1017

[33] Sakamoto T, Iwata K, Ando M (2002) Growth hormone and prolactin expression during environmental adaptation of gobies. Fish Sci 68: 757–760.

[34] Fishman AP, Galante RJ, Winokur A, Pack AI (1992) Estivation in the African Lungfish. P Am Philos Soc 136: 61–72.

[35] Holl RW, Hartman ML, Veldhuis JD, Taylor WM, Thorner MO (1991) Thirty-second sampling of plasma growth hormone in man: correlation with sleep stages. J Clin Endocrinol Metab 72: 854–861. 2005213 10.1210/jcem-72-4-854

[36] Nurnberger F, Pleschka K, Masson-Pevet M, Pevet P (1997) The somatostatin system of the brain and hibernation in the European hamster (*Cricetus cricetus*). Cell Tissue Res 288: 441–447. 9134858 10.1007/s004410050831

[37] Berg D, Holzmann C, Riess O (2003) 14-3-3 proteins in the nervous system. Nat Rev Neurosci 4: 752–762. 12951567 10.1038/nrn1197

[38] Monti JM, Jantos H (2008) The roles of dopamine and serotonin, and of their receptors, in regulating sleep and waking. Prog Brain Res 172: 625–646. 18772053 10.1016/S0079-6123(08)00929-1

[39] Murillo-Rodriguez E, Arias-Carrion O, Sanguino-Rodriguez K, Gonzalez-Arias M, Haro R (2009) Mechanisms of sleep-wake cycle modulation. CNS Neurol Disord Drug Targets 8: 245–253. 19689306 10.2174/187152709788921654

[40] Zeng L, Imamoto A, Rosner MR (2008) Raf kinase inhibitory protein (RKIP): a physiological regulator and future therapeutic target. Expert Opin Ther Targets 12: 1275–1287. 18781826 10.1517/14728222.12.10.1275

[41] Ojika K, Mitake S, Tohdoh N, Appel SH, Otsuka Y, et al (2000) Hippocampal cholinergic neurostimulating peptides (HCNP). Prog Neurobiol 60: 37–83. 10622376 10.1016/s0301-0082(99)00021-0

[42] Bergeron D, Beauseigle D, Bellemare G (1993) Sequence and expression of a gene encoding a protein with RNA-binding and glycine-rich domains in *Brassica napus* . Biochim Biophys Acta 1216: 123–125. 7916642 10.1016/0167-4781(93)90047-h

[43] Sakurai T, Itoh K, Higashitsuji H, Nonoguchi K, Liu Y, et al (2006) Cirp protects against tumor necrosis factor-alpha-induced apoptosis via activation of extracellular signal-regulated kinase. Biochim Biophys Acta 1763: 290–295. 16569452 10.1016/j.bbamcr.2006.02.007

[44] Saito K, Fukuda N, Matsumoto T, Iribe Y, Tsunemi A, et al (2010) Moderate low temperature preserves the stemness of neural stem cells and suppresses apoptosis of the cells via activation of the cold-inducible RNA binding protein. Brain Res 1358: 20–29. 20735994 10.1016/j.brainres.2010.08.048

[45] Pan F, Zarate J, Choudhury A, Rupprecht R, Bradley TM (2004) Osmotic stress of salmon stimulates upregulation of a cold inducible RNA binding protein (CIRP) similar to that of mammals and amphibians. Biochimie 86: 451–461. 15308334 10.1016/j.biochi.2004.06.006

[46] Chichiarelli S, Ferraro A, Altieri F, Eufemi M, Coppari S, et al (2007) The stress protein ERp57/GRP58 binds specific DNA sequences in HeLa cells. J Cell Physiol 210: 343–351. 17061245 10.1002/jcp.20824

[47] Erickson RR, Dunning LM, Olson DA, Cohen SJ, Davis AT, et al (2005) In cerebrospinal fluid ER chaperones ERp57 and calreticulin bind beta-amyloid. Biochem Biophys Res Commun 332: 50–57. 15896298 10.1016/j.bbrc.2005.04.090

[48] Eschenfeldt WH, Berger SL (1986) The human prothymosin alpha gene is polymorphic and induced upon growth stimulation: evidence using a cloned cDNA. Proc Natl Acad Sci USA 83: 9403–9407. 3467312 10.1073/pnas.83.24.9403 PMC387146

[49] Clinton M, Graeve L, el-Dorry H, Rodriguez-Boulan E, Horecker BL (1991) Evidence for nuclear targeting of prothymosin and parathymosin synthesized in situ. Proc Natl Acad Sci USA 88: 6608–6612. 1862085 10.1073/pnas.88.15.6608 PMC52136

[50] Manrow RE, Sburlati AR, Hanover JA, Berger SL (1991) Nuclear targeting of prothymosin alpha. J Biol Chem 266: 3916–3924. 1899869

[51] Gomez-Marquez J, Segade F, Dosil M, Pichel JG, Bustelo XR, et al (1989) The expression of prothymosin alpha gene in T lymphocytes and leukemic lymphoid cells is tied to lymphocyte proliferation. J Biol Chem 264: 8451–8454. 2785990

[52] Evstafieva AG, Belov GA, Rubtsov YP, Kalkum M, Joseph B, et al (2003) Apoptosis-related fragmentation, translocation, and properties of human prothymosin alpha. Exp Cell Res 284: 211–223. 12651154 10.1016/s0014-4827(02)00047-2

[53] Jiang X, Kim HE, Shu H, Zhao Y, Zhang H, et al (2003) Distinctive roles of PHAP proteins and prothymosin-alpha in a death regulatory pathway. Science 299: 223–226. 12522243 10.1126/science.1076807

[54] Rodriguez P, Vinuela JE, Alvarez-Fernandez L, Buceta M, Vidal A, et al (1998) Overexpression of prothymosin alpha accelerates proliferation and retards differentiation in HL-60 cells. Biochem J 331 (Pt 3): 753–761. 10.1042/bj3310753 PMC1219414 9560301

[55] Wu CL, Shiau AL, Lin CS (1997) Prothymosin alpha promotes cell proliferation in NIH3T3 cells. Life Sci 61: 2091–2101. 9395250 10.1016/s0024-3205(97)00882-5

[56] Sburlati AR, Manrow RE, Berger SL (1991) Prothymosin alpha antisense oligomers inhibit myeloma cell division. Proc Natl Acad Sci USA 88: 253–257. 1986372 10.1073/pnas.88.1.253 PMC50788

[57] Rodriguez P, Vinuela JE, Alvarez-Fernandez L, Gomez-Marquez J (1999) Prothymosin alpha antisense oligonucleotides induce apoptosis in HL-60 cells. Cell Death Differ 6: 3–5. 10200541 10.1038/sj.cdd.4400450

[58] Dominguez F, Magdalena C, Cancio E, Roson E, Paredes J, et al (1993) Tissue concentrations of prothymosin alpha: a novel proliferation index of primary breast cancer. Eur J Cancer 29A: 893–897. 8387320 10.1016/s0959-8049(05)80433-2

[59] Tsitsiloni OE, Stiakakis J, Koutselinis A, Gogas J, Markopoulos C, et al (1993) Expression of alpha-thymosins in human tissues in normal and abnormal growth. Proc Natl Acad Sci USA 90: 9504–9507. 8415730 10.1073/pnas.90.20.9504 PMC47597

[60] Karetsou Z, Kretsovali A, Murphy C, Tsolas O, Papamarcaki T (2002) Prothymosin alpha interacts with the CREB-binding protein and potentiates transcription. EMBO Rep 3: 361–366. 11897665 10.1093/embo-reports/kvf071 PMC1084059

[61] Martini PG, Delage-Mourroux R, Kraichely DM, Katzenellenbogen BS (2000) Prothymosin alpha selectively enhances estrogen receptor transcriptional activity by interacting with a repressor of estrogen receptor activity. Mol Cell Biol 20: 6224–6232. 10938099 10.1128/mcb.20.17.6224-6232.2000 PMC86097

[62] Vareli K, Frangou-Lazaridis M, van der Kraan I, Tsolas O, van Driel R (2000) Nuclear distribution of prothymosin alpha and parathymosin: evidence that prothymosin alpha is associated with RNA synthesis processing and parathymosin with early DNA replication. Exp Cell Res 257: 152–161. 10854063 10.1006/excr.2000.4857

[63] Zheng L, Roeder RG, Luo Y (2003) S phase activation of the histone H2B promoter by OCA-S, a coactivator complex that contains GAPDH as a key component. Cell 114: 255–266. 12887926 10.1016/s0092-8674(03)00552-x

[64] Hara MR, Agrawal N, Kim SF, Cascio MB, Fujimuro M, et al (2005) S-nitrosylated GAPDH initiates apoptotic cell death by nuclear translocation following Siah1 binding. Nat Cell Biol 7: 665–674. 15951807 10.1038/ncb1268

[65] Tisdale EJ, Artalejo CR (2007) A GAPDH mutant defective in Src-dependent tyrosine phosphorylation impedes Rab2-mediated events. Traffic 8: 733–741. 17488287 10.1111/j.1600-0854.2007.00569.x PMC3775588

[66] Fishman AP, Pack AI, Delaney RG, Galante RJ (1986) Estivation in *Protopterus*. J Morpho Suppl 1: 237–248.

[67] Warner JR (1999) The economics of ribosome biosynthesis in yeast. Trends Biochem Sci 24: 437–440. 10542411 10.1016/s0968-0004(99)01460-7

[68] Mathews MB, Sonenberg N, Hershey JWB (2000) Origins and principles of translational control. In: Sonenberg N, Hershey JWB, Mathews MB, editors. Translational control of gene expression: Cold Spring Harbor Laboratory Press, New York. 1–32.

[69] Rudra D, Warner JR (2004) What better measure than ribosome synthesis? Genes Dev 18: 2431–2436. 15489289 10.1101/gad.1256704

[70] Hay RT (2005) SUMO: a history of modification. Mol Cell 18: 1–12. 15808504 10.1016/j.molcel.2005.03.012

[71] Seufert W, Futcher B, Jentsch S (1995) Role of a ubiquitin-conjugating enzyme in degradation of S- and M-phase cyclins. Nature 373: 78–81. 7800043 10.1038/373078a0

[72] Tournebize R, Andersen SS, Verde F, Doree M, Karsenti E, et al (1997) Distinct roles of PP1 and PP2A-like phosphatases in control of microtubule dynamics during mitosis. EMBO J 16: 5537–5549. 9312013 10.1093/emboj/16.18.5537 PMC1170186

[73] Fong NM, Jensen TC, Shah AS, Parekh NN, Saltiel AR, et al (2000) Identification of binding sites on protein targeting to glycogen for enzymes of glycogen metabolism. J Biol Chem 275: 35034–35039. 10938087 10.1074/jbc.M005541200

[74] Korsgaard B, Mommsen TP, Wright PA (1995) Urea excretion in teleostean fishes: adaptive relationships to environment, ontogenesis and viviparity. In: Walsh PJ, Wright PA, editors. Nitrogen metabolism and excretion: CRC press, Boca Raton. 259–287.

[75] Ip YK, Chew SF, Randall DJ (2001) Ammonia toxicity, tolerance, and excretion. In: Wright PA, Anderson PM, editors. Fish Physiology, Vol 20, *Nitrogen excretion*: Academic Press, San Diego. 109–148.

[76] Webb JT, Brown GW (1976) Some properties and occurrence of glutamine synthetase in fish. Comp Biochem Physiol B 54: 171–175. 5230 10.1016/0305-0491(76)90077-8

[77] Chakravorty J, Saha N, Ratha BK (1989) A unique pattern of tissue distribution and subcellular localization of glutamine synthetase in a freshwater air-breathing teleost, *Heteropneustes fossilis* (Bloch). Biochem Int 19: 519–527.

[78] Peng KW, Chew SF, Lim CB, Kuah SSL, Kok WK, et al (1998) The mudskippers *Periophthalmodon schlosseri* and *Boleophthalmus boddaerti* can tolerate environmental NH_3_ concentrations of 446 and 36 µM, respectively. Fish Physiol Biochem 19: 59–69.

[79] Wang YX, Walsh PJ (2000) High ammonia tolerance in fishes of the family Batrachoididae (Toadfish and Midshipmen). Aquat Toxicol 50: 205–219. 10958955 10.1016/s0166-445x(99)00101-0

[80] Murray BW, Busby ER, Mommsen TP, Wright PA (2003) Evolution of glutamine synthetase in vertebrates: multiple glutamine synthetase genes expressed in rainbow trout (*Oncorhynchus mykiss*). J Exp Biol 206: 1511–1521. 12654890 10.1242/jeb.00283

[81] Wright PA, Steele SL, Huitema A, Bernier NJ (2007) Induction of four glutamine synthetase genes in brain of rainbow trout in response to elevated environmental ammonia. J Exp Biol 210: 2905–2911. 17690239 10.1242/jeb.003905

[82] Lindell SL, Klahn SL, Piazza TM, Mangino MJ, Torrealba JR, et al (2005) Natural resistance to liver cold ischemia-reperfusion injury associated with the hibernation phenotype. Am J Physiol Gastrointest Liver Physiol 288: G473–G480. 15701622 10.1152/ajpgi.00223.2004

[83] Dave KR, Prado R, Raval AP, Drew KL, Perez-Pinzon MA (2006) The arctic ground squirrel brain is resistant to injury from cardiac arrest during euthermia. Stroke 37: 1261–1265. 16574920 10.1161/01.STR.0000217409.60731.38

[84] Christian SL, Ross AP, Zhao HW, Kristenson HJ, Zhan X, et al (2008) Arctic ground squirrel (*Spermophilus parryii*) hippocampal neurons tolerate prolonged oxygen-glucose deprivation and maintain baseline ERK1/2 and JNK activation despite drastic ATP loss. J Cereb Blood Flow Metab 28: 1307–1319. 18398417 10.1038/jcbfm.2008.20 PMC2792705

[85] Murakami K, Kondo T, Epstein CJ, Chan PH (1997) Overexpression of CuZn-superoxide dismutase reduces hippocampal injury after global ischemia in transgenic mice. Stroke 28: 1797–1804. 9303028 10.1161/01.str.28.9.1797

[86] Shan X, Chi L, Ke Y, Luo C, Qian S, et al (2007) Manganese superoxide dismutase protects mouse cortical neurons from chronic intermittent hypoxia-mediated oxidative damage. Neurobiol Dis 28: 206–215. 17719231 10.1016/j.nbd.2007.07.013 PMC2100412

[87] Jang YC, Perez VI, Song W, Lustgarten MS, Salmon AB, et al (2009) Overexpression of Mn superoxide dismutase does not increase life span in mice. J Gerontol A Biol Sci Med Sci 64: 1114–1125. 19633237 10.1093/gerona/glp100 PMC2759571

[88] Zemlyak I, Brooke SM, Singh MH, Sapolsky RM (2009) Effects of overexpression of antioxidants on the release of cytochrome c and apoptosis-inducing factor in the model of ischemia. Neurosci Lett 453: 182–185. 19429031 10.1016/j.neulet.2009.02.020

[89] Page MM, Salway KD, Ip YK, Chew SF, Warren SA, et al (2010) Upregulation of intracellular antioxidant enzymes in brain and heart during estivation in the African lungfish *Protopterus dolloi* . J Comp Physiol B 180: 361–369. 19888582 10.1007/s00360-009-0416-7

[90] Rostovtseva T, Colombini M (1996) ATP flux is controlled by a voltage-gated channel from the mitochondrial outer membrane. J Biol Chem 271: 28006–28008. 8910409 10.1074/jbc.271.45.28006

[91] Pfeffer SR, Rothman JE (1987) Biosynthetic protein transport and sorting by the endoplasmic reticulum and Golgi. Annu Rev Biochem 56: 829–852. 3304148 10.1146/annurev.bi.56.070187.004145

[92] Pryer NK, Wuestehube LJ, Schekman R (1992) Vesicle-mediated protein sorting. Annu Rev Biochem 61: 471–516. 1497318 10.1146/annurev.bi.61.070192.002351

[93] Rothman JE, Orci L (1992) Molecular dissection of the secretory pathway. Nature 355: 409–415. 1734280 10.1038/355409a0

[94] Serafini T, Orci L, Amherdt M, Brunner M, Kahn RA, et al (1991) ADP-ribosylation factor is a subunit of the coat of Golgi-derived COP-coated vesicles: a novel role for a GTP-binding protein. Cell 67: 239–253. 1680566 10.1016/0092-8674(91)90176-y

[95] Serafini T, Stenbeck G, Brecht A, Lottspeich F, Orci L, et al (1991) A coat subunit of Golgi-derived non-clathrin-coated vesicles with homology to the clathrin-coated vesicle coat protein beta-adaptin. Nature 349: 215–220. 1898984 10.1038/349215a0

[96] Smith MM (1991) Histone structure and function. Curr Opin Cell Biol 3: 429–437. 1892654 10.1016/0955-0674(91)90070-f

[97] Alberts B, Johnson A, Lewis J, Raff M, Roberts K, et al.. (2007) Molecular biology of the cell, 5th edn: Garland Sciences, New York.

[98] Fischle W, Wang Y, Allis CD (2003) Histone and chromatin cross-talk. Curr Opin Cell Biol 15: 172–183. 12648673 10.1016/s0955-0674(03)00013-9

[99] Wei Y, Mizzen CA, Cook RG, Gorovsky MA, Allis CD (1998) Phosphorylation of histone H3 at serine 10 is correlated with chromosome condensation during mitosis and meiosis in *Tetrahymena* . Proc Natl Acad Sci USA 95: 7480–7484. 9636175 10.1073/pnas.95.13.7480 PMC22657

[100] Gurley LR, D’Anna JA, Barham SS, Deaven LL, Tobey RA (1978) Histone phosphorylation and chromatin structure during mitosis in Chinese hamster cells. Eur J Biochem 84: 1–15. 206429 10.1111/j.1432-1033.1978.tb12135.x

[101] Krishnan S, Horowitz S, Trievel RC (2011) Structure and function of histone H3 lysine 9 methyltransferases and demethylases. Chembiochem 12: 254–263. 21243713 10.1002/cbic.201000545

[102] Luo Y, Jian W, Stavreva D, Fu X, Hager G, et al (2009) Trans-regulation of histone deacetylase activities through acetylation. J Biol Chem 284: 34901–34910. 19822520 10.1074/jbc.M109.038356 PMC2787352

